# Department of Dermatology

**Published:** 1893-04

**Authors:** Isadore Dyer

**Affiliations:** Tulane University, New Orleans


### DEPARTMENT OF DERMATOLOGY.

[Notes by Dr. Isadore Dyer, Tulane University, N. O.]

---

### TRAITMENT DE LA SYPHILIS—RUEFF & CIE., PARIS, 1893.

*Treatment of Syphilis—Prof. Alfred Fournier.*

This work has just appeared in French. Although there is nothing absolutely new in it, it would be difficult to imagine a clearer exposition of the modern views on the subject. All theories and methods are discussed, weighed and condemned in place. It is a thoroughly logical view of the different methods suggested for the treatment of syphilis, with a final statement of

Prof. Fournier's own views. Adapted from his lectures at the Saint Louis Hospital in Paris; the style in conversational, and though fulfilling excellently the work of a text book, it is an interesting work as well. The abortion of syphilis, the use of animal serum in the treatment, and the discussion of the various remedies used have each a chapter full of interest. Of mercurials the author prefers the prot-iodides and the bi-chloride. Mercury, he believes should be administered in full therapeutic doses. The intermittent treatment is advocated while opportunistic or the symptomic treatment is condemned as not fulfilling the indications. Chronic diseases should be treated in a chronic way, and syphilis should be treated by prolonged medication. Prof. Fournier administers mercury in courses of six weeks each, with corresponding intervals of rest, over a period of two or three years. Then, and not till then, the iodide, (sodium or potassium), is begun and for the first year, is given for about six weeks at a time, with intervals of rest. The second year of the iodide, the medicine is given in only three courses of six weeks each, and the third year in two courses of like length. The author believes in the cumulative action of both drugs, and favors intervals of rest on this account first, and again because it prevents the patient growing accustomed to the drug. The iodide he prefers to give in good doses and in continued doses, to the increasing dose method.

No detail is omitted in making this a thorough investigation of all methods, while arguments based on long clinical experience defend the methods approved.

Diet and hygiene find full notice. The neurasthenia of syphilis, indications for treatment, with hydrotheraphy, all find room.

The work commends itself, and it can hardly be long before an English translation will be afforded those who are interested. The cleanliness of its discussions and the rational suggestions with which the book is filled will find favor with both student and practitioner.

---

DISEASES OF THE SKIN—DR. C. C. RANSOM, NEW YORK.—STUDENTS' QUIZ SERIES—LEA BROTHERS & CO., PHILADELPHIA, 1893.

There is a certain originality in this little book which makes it useful. Diseases of the skin are handled in a clear and concise way. Space is economized, while material does not suffer. The definitions are excellent. The alphabetical arrangement, and the formulary in the back of the book, will make it welcome to the busy physician. The treatment is suggested with judgment and the views of treatment are liberal. Dr. Ransom's connection with the Vanderbilt Chair in New York, has afforded him ample material to make this little book quite practical.

